# Determining the cost-effectiveness requirements of an exoskeleton preventing second hip fractures using value of information

**DOI:** 10.1186/s12913-020-05768-4

**Published:** 2020-10-15

**Authors:** Stefania Manetti, Giuseppe Turchetti, Francesco Fusco

**Affiliations:** 1 Institute of Management, Scuola Superiore Sant’Anna, Pisa, Italy; 2 Department of Surgery and Cancer, St Mary’s Hospital, Imperial College London, London, UK; 3 Health Economics Research Centre, Nuffield Department of Population Health, University of Oxford, Old Road Campus, Headington, Oxford, UK; 4 Centre for Health Economics, University of York, Heslington, York, UK; 5 Department of Public Health & Primary Care, University of Cambridge, Institute of Public Health, Forvie Site, Robinson Way, Cambridge, CB2 0SR UK

**Keywords:** Cost-effectiveness analysis, Early health technology assessment, Value of information, Uncertainty, Elicitation

## Abstract

**Background:**

Falls may lead to hip fractures, which have a detrimental effect on the prognosis of patients as well as a considerable impact on healthcare expenditures. Since a secondary hip fracture (SHF) may lead to even higher costs than primary fractures, the development of innovative services is crucial to limit falls and curb costs in high-risk patients. An early economic evaluation assessed which patients with a second hip fracture could benefit most from an exoskeleton preventing falls and whether its development is feasible.

**Methods:**

The life-course of hip fractured patients presenting with dementia or cardiovascular diseases was simulated using a Markov model relying on the United Kingdom administrative data and complemented by published literature. A group of experts provided the exoskeleton parameters. Secondary analyses included a threshold analysis to identify the exoskeleton requirements (e.g. minimum impact of the exoskeleton on patients’ quality of life) leading to a reimbursable incremental cost-effectiveness ratio. Similarly, the uncertainty around these requirements was modelled by varying their standard errors and represented alongside population Expected Value of Perfect Information (EVPI).

**Results:**

Our base-case found the exoskeleton cost-effective when providing a statistically significant reduction in SHF risk. The secondary analyses identified 286 cost-effective combinations of the exoskeleton requirements. The uncertainty around these requirements was explored producing further 22,880 scenarios, which showed that this significant reduction in SHF risk was not necessary to support the exoskeleton adoption in clinical practice. Conversely, a significant improvement in women quality of life was crucial to obtain an acceptable population EVPI regardless of the cost of the exoskeleton.

**Conclusions:**

Our study identified the exoskeleton requisites to be cost-effective and the value of future research. Decision-makers could use our analyses to assess not only whether the exoskeleton could be cost-effective but also how much further research and development of the exoskeleton is worth to be pursued.

## Background

Falls have a detrimental impact on mortality, morbidity and Health-Related Quality of Life (HRQOL) since they may lead to traumatic events, such as hip fractures [1–3]. In the United Kingdom (UK), the latest estimates of the health and social costs of hip fractures range from £2 to £3 billion. These costs are expected to increase further by 2025 mainly because of osteoporosis-related fractures [2, 4–6]. Secondary hip fracture (SHF) may lead to even larger costs than primary fractures, mainly due to the likely increase in duration of hospitalisation [2, 7]. Some comorbidities, such as dementia and cardiovascular disease (CVD), are known to impair the gait and as such are important risk factors for SHFs [8]. Therefore, there is an increasing interest in the identification of cost-effective interventions to prevent falls, boosting research and development of innovative technologies in this area [9–12].

A recent study suggested that robotic exoskeletons could avoid falls, but more evidence would be needed to decide whether it would be a cost-effective intervention to prevent SHFs [13]. Given the limited evidence on the exoskeleton effectiveness, traditional Health Technology Assessment (HTA) methods would not be sufficient to assess the device cost-effectiveness. Nevertheless, an economic evaluation could be performed by using early HTA approach. Early HTA is defined as the collection of methods used to inform decision makers, and manufacturers, on the possible value of a technology in development and includes methods to manage and quantify uncertainty [14]. An intrinsic characteristic of the evaluation of technologies at their early stage is the dearth of reliable parameters, which can often only be obtained by use of elicitation methods. Decision models employing these methods will provide valid estimates insofar the elicited values are confirmed by future studies. Even if elicited values are confirmed by future evidence, they do not necessarily represent the minimum requirements to ensure the cost-effectiveness of a technology. For example, elicited values do not provide information on what is the minimum average health effect, and the relative uncertainty, necessary to justify the cost of an intervention. A sequence of threshold analyses could identify these requirements as well as express their uncertainty in terms Value of Information (VOI). VOI is used in decision analysis to represent simultaneously the probability that a decision will be wrong and its consequences in the same terms of value as the decision being made [15]. Then, VOI could be interpreted as the upper limit to fund additional research to resolve all uncertainty in current available evidence. Therefore, providing an estimate of the minimum cost-effectiveness requirements expressed in terms of VOI could inform not only the manufacturer but also decision-makers on whether these requirements are achievable and, more importantly, worthy of pursuance [16].

In this respect, the substantial costs to fund new research may raise important questions for the technology and its potential users. For example, (i) which subpopulation of patients may benefit most from the use of the new technology, (ii) what are, on average, the minimum requirements for the technology to be cost-effective, (iii) what is the maximum uncertainty around these requirements to justify future research, and (iv) are the values identified in (ii) and (iii) achievable? An early economic evaluation of an exoskeleton able to prevent falls is used as case study to illustrate this process. The current case study uses a sequence of threshold analyses to answer questions (i) to (iii). In doing so, this analysis will support the decision on whether the further research and development of the exoskeleton is worthwhile (i.e. question iv).

## Methods

We performed an early cost-effectiveness analysis of a robotic exoskeleton, by simulating its use both in a care-home and in patients’ home settings, in addition to UK standard post-hip fracture care [13, 17].

Our analyses are based on a previously published Markov model developed in Excel (Microsoft, Redmond, WA) [7, 17]. The model described the natural history of patients with hip fractures and, by doing so, estimated the life-expectancy trajectories, allowing us to calculate the lifetime costs and Quality Adjusted Life Years (QALYs) of the simulated patients. These estimates were conditional on health states reflecting the natural history of hip fracture: history of primary hip fracture, second hip fracture and major non-hip fractures (e.g. wrist, spine, humerus) necessitating hospitalisation. These states were implemented both in a care-home and own home settings. Death was modelled as 30-day mortality after a fracture or within a year. Once patients were moved to a care-home, they were not modelled to return to their own home. The cycle length used was one year, applying a half-cycle correction. A 3.5% annual discount rate was applied to costs (2012/2013 UK sterling) and to QALYs [17]. The model schematic is depicted in the Additional file 1: Appendix 1.

### Target population

The population simulated in our model reflects the characteristics of the UK population who had experienced a hip fracture observed between 2003 and 2013. Our analyses focused on high-risk subpopulations and included patients with dementia and patients with CVD (i.e. stroke, myocardial infarction) [7, 8]. The subgroups explored in our analyses were sex and age specific risks at 65, 75 and 85 years old.

### Model parameters

The model was parameterised using an extract of the Hospital Episode Statistics (HES) dataset and supplemented by published literature [7, 17]. This extract included 33,152 individuals older than 60 years who had an emergency admission for a hip fracture coded with an ICD-10 diagnosis code S72.0- S72.2 or S72.9. These data informed the model on the probabilities of having a fracture, being discharged to a care-home and dying. Similarly, HES data were used to calculate the cost of hospitalisations relative to the model health states. Information on primary care visits, drug consumption and laboratory tests were obtained from an extract of the Clinical Practice Research Datalink (CPRD) dataset. This extract included 4063 patients who had a link to hospital admission due to a hip fracture and was used to estimate the primary care costs for each health state in the model. Additional file 1: Appendices 2 and 3 show the risk equations and events probabilities that informed the model. More detail of our methodology has been previously reported [7, 17].

Subpopulations were modelled by adjusting the standard population mortality and the probability of having a SHF. The equations estimating mortality at 30 days after an SHF surgery used as a covariate the Charlson Comorbidity Index (CCI). The 30-day mortality was calculated assuming a score of 2.2 for both the CVD and dementia subpopulations. The probability of having a second hip fracture in dementia and CVD subpopulation was updated using odds ratios from a previously published meta-analysis, respectively 1.89 (95% confidence interval [95% CI]: 1.47 to 2.43) and 1.32 (95% CI: 1.02 to 1.70) [8]. Additional file 1: Appendix 4 shows the parameters used to model the subpopulations.

### Quality of life

A meta-regression, employing a mixed-effect model, estimated the utilities obtained from 21,085 patients from 32 studies assessing preference-based quality of life for individuals with hip fracture. This model was used to predict the utility values of non-comorbid population patients and was assumed to remain constant after the first year following a hip fracture (0.66). The impact of a second hip fracture and a major non-hip fracture on the HRQOL were assumed to be comparable and, therefore, were represented by the same utility (0.44) [17]. Additional file 1: Appendix 5 shows the coefficient of the model used to calculate the utility scores.

The disutilities of the CVD (ICD-9 codes 412 and 433) and dementia (ICD-9 code 331) subpopulations were obtained from Sullivan et al. [18]. These disutilities were modelled as a decrement relative to the general population and, therefore, the utilities were reduced by 4% for CVD patients and 26% for dementia patients.

### Costs

The perspective of the National Health Service (NHS) in England and personal social services (PSS) was adopted, including primary and secondary healthcare and care-home costs. Costs considered are those both related and unrelated to hip fractures. Primary care expenditures were comprised of practice nurse and GP contacts, as well as visits to other healthcare personnel, such as health visitors or physiotherapists, drugs and laboratory tests. Secondary healthcare costs included accident and emergency contacts, outpatient visits, inpatient admissions and day cases. The cost of walking aids, home adaptations or home care were not included since these are funded by councils or local organisations. Comorbidities were found to be an important predictor for the cost of primary hip fracture, but not for secondary (or later) fractures [7]. Therefore, we assumed the costs due to SHFs in dementia and CVD subpopulations were comparable to the population without comorbidities. Additional file 1: Appendices 6–8 show the equations used to calculate the costs that informed the decision model.

### Exoskeleton parameters

The parameters used in the base-case, and their respective uncertainty, were obtained through semi-structured interviews of experts. The group of experts composed of the developers of the exoskeleton and a senior orthopaedics consultant of a hospital (i.e. ASL Nord-Ovest) in Tuscany (Italy). The engineers expected their exoskeleton to decrease the number of falls leading to SHFs by 25%. This reduction was modelled as a hazard ratio (HR), and the standard error was assumed to be 40% of this estimate on the logarithmic scale. The senior consultant found this HR plausible and believed this would increase the patients’ HRQOL by 70%. The additional uncertainty around the HRQOL due to the device was incorporated in the model inflating the utility estimates of the subpopulations by 40%. The base-case assumed that the exoskeleton was provided to the NHS at an annual cost of £6000, including servicing costs. Additional file 1: Appendix 4 shows the exoskeleton parameters.

### Analysis

A probabilistic sensitivity analysis was performed to estimate the uncertainty around the costs and QALYs in each group. The convergence of the model was tested by considering an increasing number of simulations until a stable estimate of costs and QALYs was obtained. In doing so, it was determined that the model converged using 1000 simulations. Based on these simulations, we calculated the between groups differences in costs and QALYs. The uncertainty around these mean differences was reported using a non-parametric 95% CI. The difference in costs was combined with the difference in QALYs to calculate the Incremental Cost-Effectiveness Ratio (ICER) of the exoskeleton. To assess the value for money of the exoskeleton, ICERs were compared to a cost-effectiveness threshold of £20,000 per QALY [19]. Assuming that this estimate represents the actual monetary value of a QALY allowed us to calculate the expected value of perfect information (EVPI) for the UK population. To calculate the population EVPI, the cumulative number of hip fractured patients, with either CVD or dementia, in the next 10 years was multiplied by the EVPI [7, 20]. Our base-case results are presented by sex, comorbidity and age bands, namely: 65, 75, and 85 years of age. Secondary analyses were performed in two steps, namely threshold and uncertainty analyses.

### Threshold analysis

The threshold analysis aimed at answering questions (i) and (ii) outlined in the background. To identify which patients may benefit most from the use of the exoskeleton (i.e. question (i)), the average minimum requirements for the technology to be cost-effective (i.e. question (ii)) were identified for each subgroup.

**Fig. 1:**
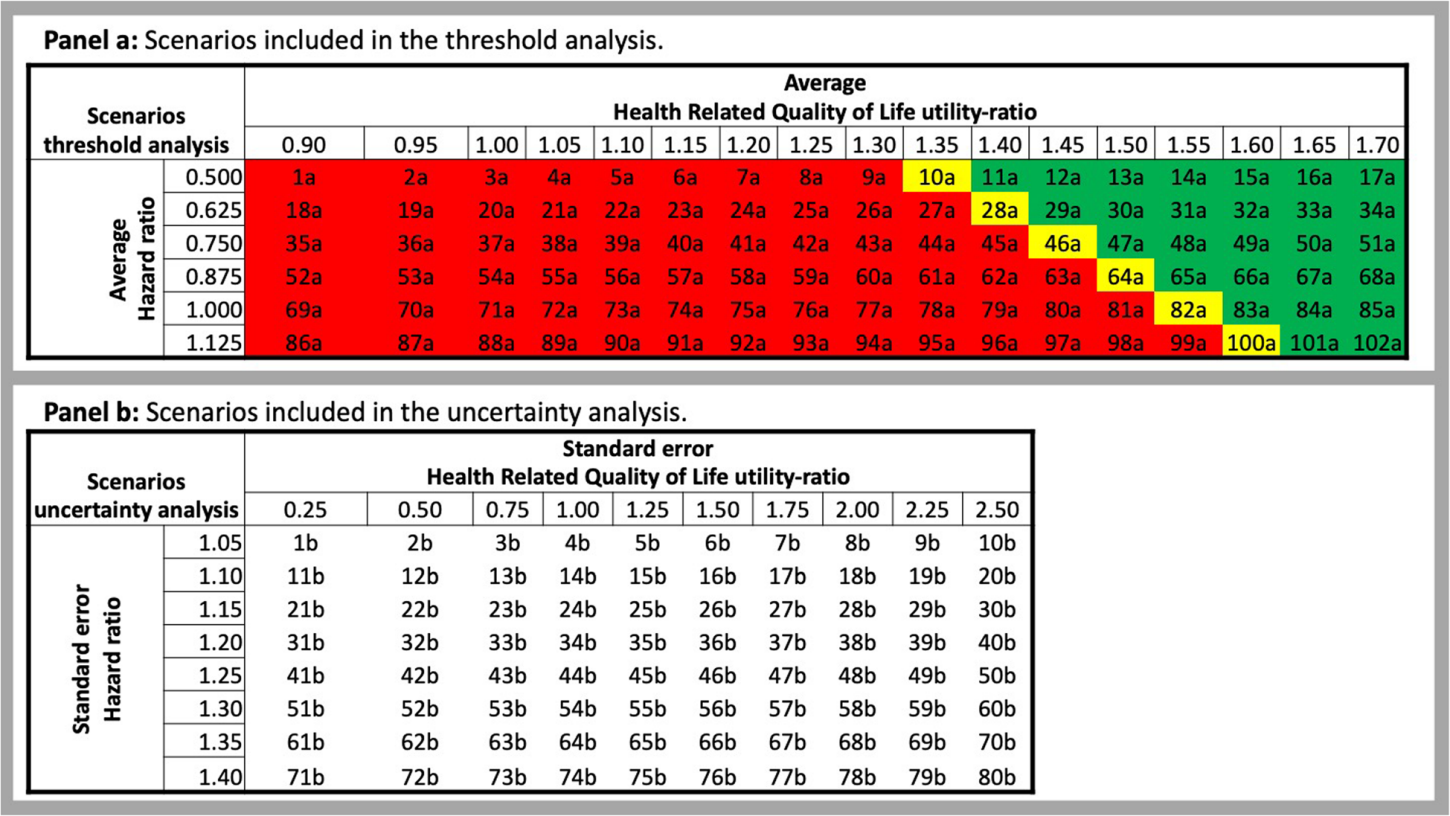
Schematic of the secondary analyses: (**a**) Threshold analysis, (**b**) Uncertainty analysis

The threshold analysis assumed that there was no uncertainty around the parameters of the exoskeleton. In this respect, the value of the HR of falling of patients wearing the exoskeleton and their HRQOL utility-ratio were kept constant (i.e. deterministic) over the 1000 simulation of each scenario. The scenarios included in the threshold analysis were obtained by combining arbitrary values of HR of falling wearing the exoskeleton to values of the HRQOL of the patients using the device. The HR of falling ranged from 0.5 to 1.125, and the intermediate scenarios were based on intervals of 0.125. Similarly, the HRQOL of the patients wearing the device was adjusted by utility-ratios ranging arbitrarily from 0.90 to 1.70, using intervals of 0.05. Figure 1 (panel a) depicts these scenarios.

For example, scenario 1a assumed that the HR of falling with the exoskeleton is 0.5 and the utility-ratio is equal to 0.90. Based on these values, a thousand simulations were obtained and stored for scenario 1a. Then, it was assumed that the HR of falling was still equal to 0.5 and the utility-ratio was increased from 0.90 to 0.95 to obtain scenario 2a. Based on this new set of values, 1000 runs were simulated and recorded for scenario 2a. These steps are repeated until the largest utility-ratio considered in our analysis was combined with the HR of falling equal to 0.5 (i.e. scenario 17a). This process was performed for the other scenarios of the HR of falling, namely assuming that the HR of falling is equal to 0.625, 0.75, 0.875, 1, or 1.125.

This iterative approach was performed for all the considered age bands (i.e. 65, 75 and 85 years), sex, comorbidities (i.e. CVD, dementia), risk-sharing agreement (i.e. leasing or purchase) and the respective cost of the device (leasing cost: £3000, £4000, £5000 and £6000; purchase cost: £12,500, £15,000, £17,500 and £20,000). The ‘purchase’ scenarios assumed that costs were irreversible, the exoskeleton lifespan was 5-years and the annual maintenance costed £2500 per year [21]. As a result of this process, 9792 scenarios were obtained and 1000 simulations were stored for each scenario. All the 1000 simulations were used to calculate an ICER per scenario. The scenarios providing an ICER below £20,000 per QALY, which are shaded in green and yellow in Fig. 1 (panel a), were retained and further analysed. Based on the considered discrete values of the HR of falling, the scenarios presenting the lowest utility-ratio but still leading to an ICER below £20,000 per QALY (i.e. yellow shaded scenarios in Fig. 1; panel a) represent the exoskeleton requirements to be cost-effective. These requirements were employed in the uncertainty analysis.

### Uncertainty analysis

The exoskeleton requirements obtained in the threshold analysis were used to inform the uncertainty analysis, which aimed at answering question (iii) reported in the background. The uncertainty around these requirements was modelled in 80 further scenarios, which were obtained by combining arbitrary values of the standard errors of the exoskeleton requirements. The standard error applied to the exoskeleton HR of falling ranged from 1.05 to 1.40, employing intervals of 0.05. The standard error of the HRQOL utility-ratio was increased from 0.25 to 2.5 by intervals of 0.25. The uncertainty of the exoskeleton HR and utility-ratio were modelled with a log-normal distribution. All possible combinations of the standard errors outlined above were combined and presented in in Fig. 1 (panel b). The overall model uncertainty of each scenario was then quantified by calculating the population EVPI.

For example, assuming that the threshold analysis determined that the exoskeleton is cost-effective if the average HR of falling is equal to 0.5 and the average utility-ratio if equal to 1.35 (i.e. scenario 10a of Fig. 1; panel a), the standard errors around these values were varied in 80 scenarios. In this sense, the standard error of the HR of falling is initially set equal to 1.05 and the standard error of the utility-ratio is set to 0.25 (i.e. scenario 1b of Fig. 1; panel b). Based on these values of the parameters and their uncertainty (i.e. standard error), 1000 simulations were obtained and stored. Then, assuming the standard error at the HR of falling is still equal to 1.05, the standard error of the utility-ratio was increased from 0.25 to 0.5 (i.e. scenario 2b of Fig. 1; panel b) and 1000 runs were again obtained and recorded. These steps are repeated until the largest standard error considered for the utility-ratio (i.e. scenario 10b of Fig. 1; panel b) was combined with the standard error of the HR of falling equal to 1.05. The process for the considered threshold value (i.e. average HR of falling equal to 0.5 and utility-ratio equal to 1.35) ended when the steps outlined above were performed for all the scenarios of the standard errors of HR of falling, namely assuming that the standard error is equal to 1.1, 1.15, 1.2, 1.25, 1.3, 1.35 or 1.4.

This iterative approach was performed for each scenario identified in the threshold analysis – which are shaded in yellow in Fig. 1 (panel a). The stored results were used to obtain non-parametric 95% CI of the HR of falling and HRQOL utility-ratio of each scenario, and to calculate the respective population EVPI. These results were analysed using quantile regressions to describe the 95% confidence intervals of the threshold values as a function of the population EVPI. Additional file 1: Appendices 9 and 10 show the coefficients of these quantile regressions.

## Results

### Base-case

The Markov model simulated cohorts of 1000 patients to reflect the characteristics of dementia and CVD patients accessing a hospital because of a hip fracture in the UK. Table 1 shows our base-case results by sex and age bands. Our model showed that the exoskeleton could lead to a substantial increment of QALYs regardless of patients’ age and comorbidities. For patients with CVD, the exoskeleton is estimated to be cost-effective only in CVD patients younger than 75 years (ICER (female): £18,753; ICER (male): £19,598). For dementia patients, the exoskeleton is similarly cost-effective in preventing SHF only in individuals not older than 75 years. The ICER for dementia patients ranged from £18,083 (65-year-old female) to £19,900 (75-year-old male).

**Table 1:** Base-case: Lifetime costs and QALYs of cardiovascular disease and dementia hip fractured populations by sex and age

	Exoskeleton		Usual care		Difference (95% Confidence Interval)		
	Costs	QALYs	Costs	QALYs	Cost	QALYs	ICER
**Cardiovascular disease**							
Female							
65 years old	£104,735	7.26	£59,588	4.85	£45,147(£41,350 to £49,207)	2.41(1.91 to 2.97)	£18,753
75 years old	£80,067	4.40	£52,210	3.01	£27,857(£26,332 to £29,680)	1.39(1.13 to 1.63)	£20,041
85 years old	£60,162	2.44	£43,972	1.74	£16,190(£15,530 to £16,894)	0.7(0.59 to 0.8)	£23,169
Male							
65 years old	£84,062	5.28	£50,867	3.59	£33,195(£30,346 to £35,895)	1.69(1.32 to 2.09)	£19,598
75 years old	£61,070	2.97	£41,674	2.09	£19,396(£18,188 to £20,534)	0.88(0.69 to 1.04)	£22,122
85 years old	£44,656	1.53	£33,817	1.15	£10,839(£10,332 to £11,355)	0.38(0.32 to 0.44)	£28,467
**Dementia**							
Female							
65 years old	£105,299	6.14	£60,854	3.68	£44,445(£40,793 to £48,371)	2.46(1.66 to 3.2)	£18,083
75 years old	£80,461	3.74	£53,076	2.29	£27,385(£25,869 to £29,187)	1.44(1.04 to 1.81)	£18,971
85 years old	£60,384	2.10	£44,478	1.34	£15,906(£15,256 to £16,602)	0.75(0.58 to 0.93)	£21,091
Male							
65 years old	£84,393	4.51	£51,668	2.74	£32,725(£29,951 to £35,362)	1.77(1.25 to 2.31)	£18,476
75 years old	£61,303	2.57	£42,208	1.61	£19,094(£17,918 to £20,195)	0.96(0.72 to 1.19)	£19,900
85 years old	£44,801	1.36	£34,134	0.90	£10,667(£10,162 to £11,169)	0.46(0.36 to 0.56)	£23,341

The 95% CIs of the HR and HRQOL utility-ratio ranged from 0.60 to 0.94 and from 1.46 to 1.93, respectively. As a result of these uncertainties, the population EVPI of cost-effective scenarios for female patients ranged from £12,058,172 (CVD - 65 years old) to £29,986,626 (dementia - 75 years old), whilst the population EVPI for 65-year-old women with dementia was £20,332,551. The smaller proportion of male patients experiencing a hip fracture, approximately 25% of the HES extract, [7, 17] was reflected by the population EVPI, which ranged from £5,275,225 (CVD - 65 years old) to £9,920,712 (dementia - 75 years old). The population EVPI for male patients aged 65 with dementia was £5,460,316.

### Threshold analysis

**Fig. 2:**
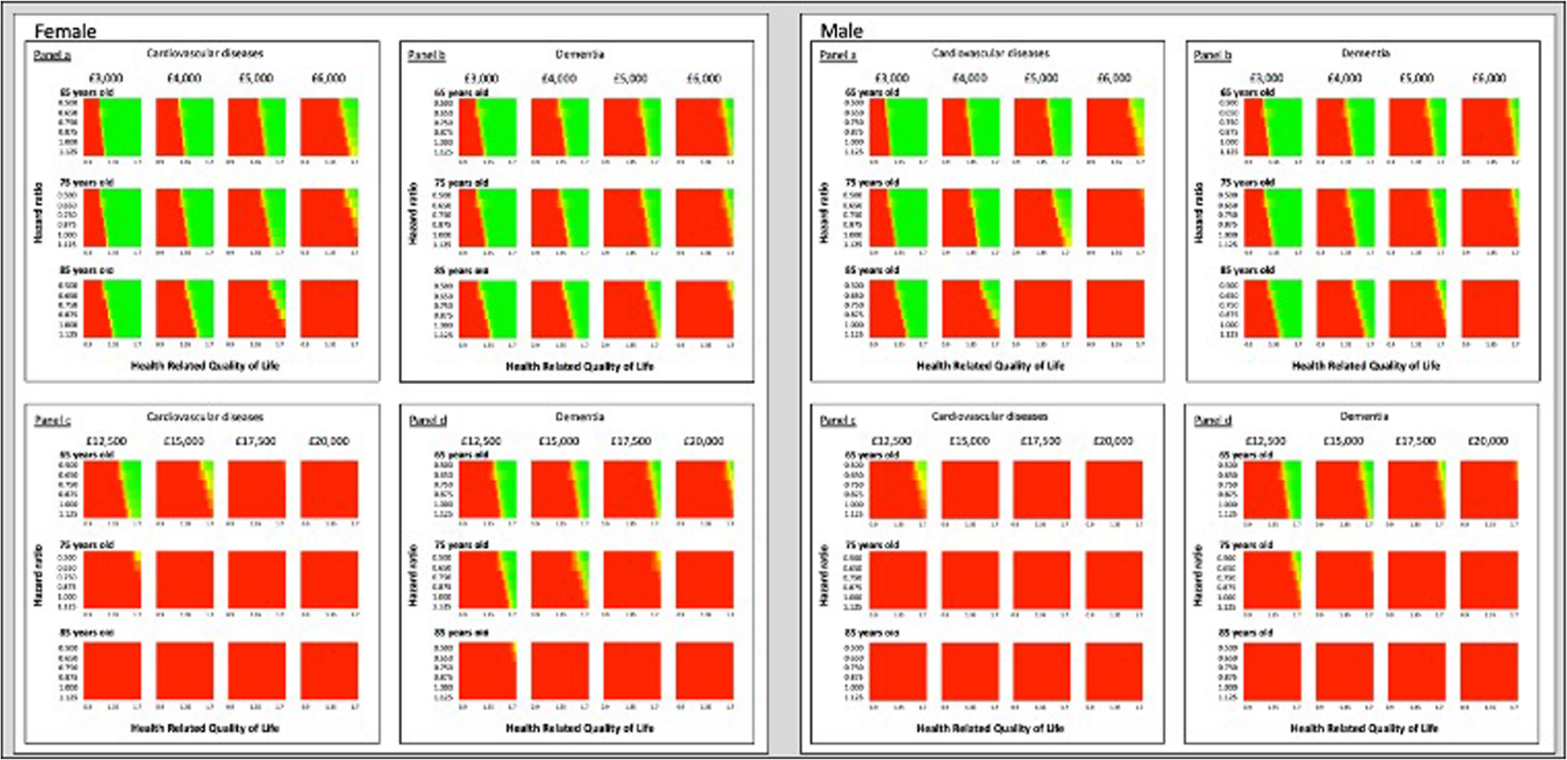
Threshold analysis: Cost-effectiveness heat map of cardiovascular and dementia hip fractured populations by sex and age. Legend: Green (cost-effectiveness probability = 1); red (cost-effectiveness probability = 0)

Figure 2 depicts the probability of cost-effectiveness of each examined scenario on a chromatic scale ranging from red (i.e. not cost-effective) to green (cost-effective). Panels A and B report the cost-effectiveness results for the “leasing” scenarios, whilst panels C and D depict the “purchase” scenarios, for CVD and dementia cohorts. The vertical axis of each figure in the panels reports the exoskeleton HR, the HRQOL utility-ratio is represented on the horizontal end.

### Leasing

The importance of HRQOL across the leasing scenarios is more prominent than fall prevention (Fig. 2; panels a and b).

The exoskeleton was not cost-effective unless it provided a gain in patients’ HRQOL by 25% paired to a HR of falling of 0.625. However, the reduction in the number of second hip fractures was not a limiting factor for cost-effectiveness, especially in less expensive scenarios. The largest HR of falling that still ensured the device’s cost-effectiveness was 1.125, however, this scenario needs to be paired with a larger increment in HRQOL, namely 30%. Conversely, the prevention of hip fractures had a relevant impact in scenarios simulating more expensive leasing fees (e.g. £6000 annual leasing). These fees could be justified only if the exoskeleton was not inferior to usual care, and would provide on average a larger improvement in patients’ HRQOL.

The proportion of cost-effective scenarios did not materially differ between disease groups, regardless of sex.

### Purchase

Compared to the leasing scenarios, purchasing the device (Fig. 2; panels c and d) was less likely to be a cost-effective alternative. In this respect, the HRQOL influences the results of the purchase scenarios to a smaller extent than it did for the leasing scenarios.

Overall, to represent good value for money, the device should provide at least an increment of patients’ HRQOL by 48% alongside an HR of falling of 50% SHF. Similarly to the leasing scenarios, the HR of falling does not influence predominantly the cost-effectiveness of the device (i.e. the threshold value is 1.25), but the increment in HRQOL will need to be substantially large, namely 57.5%.

Selling the device is more likely to be a cost-effective alternative in patients with dementia, especially in women not older than 75 years (Fig. 2; panels c and d).

### Uncertainty analysis

**Fig. 3:**
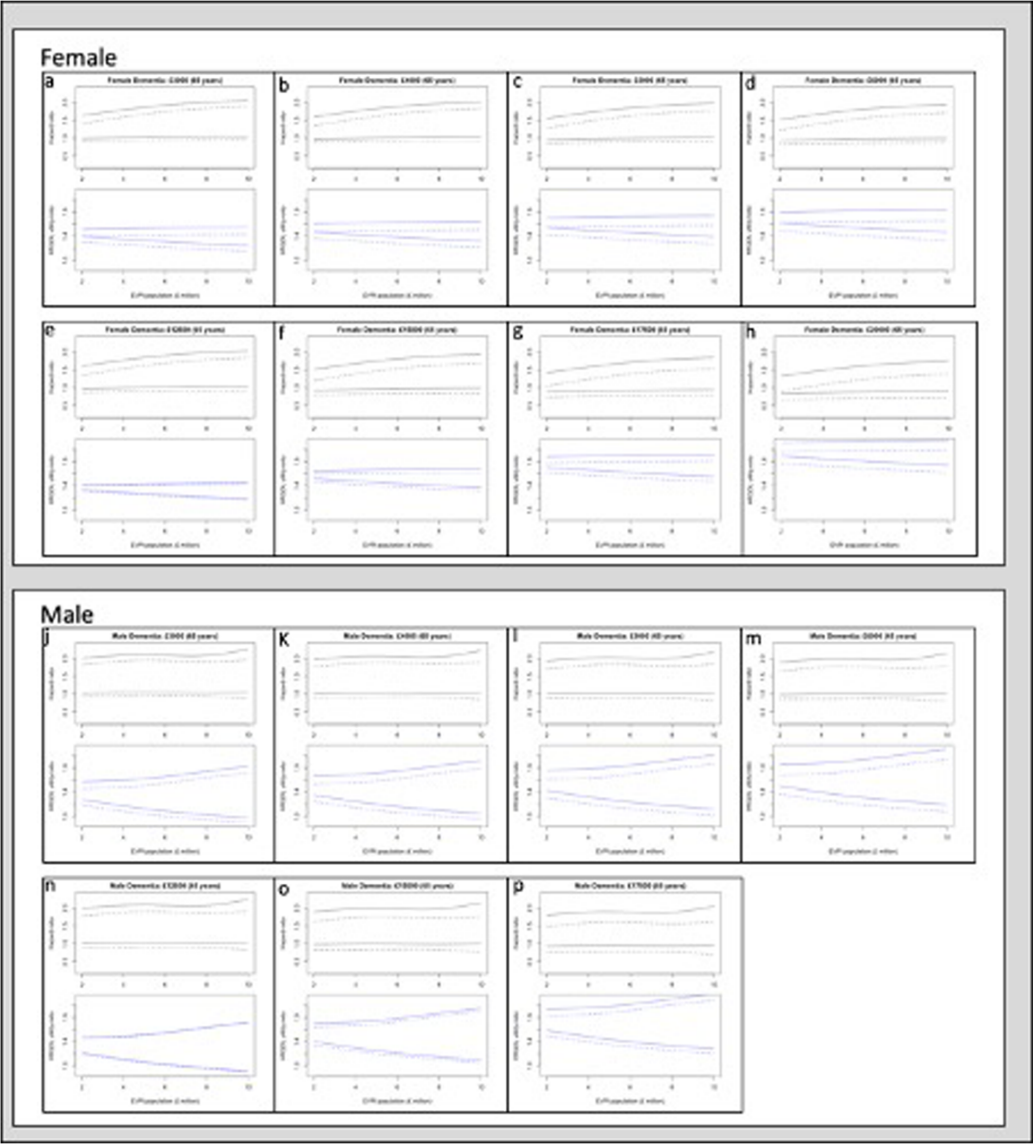
Uncertainty analysis: 95% confidence interval of HRQOL utility-ratio, 95% confidence interval of SHF hazard ratio as a function of the expected value of information at population level (£ million). Abbreviations: Health Related Quality of Life (HRQOL); Expected Value of Perfect Information (EVPI). Legend: dashed lines (HRQOL utility-ratio threshold); solid lines (hazard ratio threshold)

Figure 3 represents the uncertainty around threshold values of HR (solid lines) and HRQOL (dashed lines) in 65-year-old patients with dementia while the full set of scenarios is available at Additional file 1: Appendices 11–18. These uncertainties were plotted against a common x-axis representing the population EVPI.

### Leasing

#### Confidence intervals around the HR threshold values

The exoskeleton would be cost-effective even if it induced falls as long as it would provide a large increment in HRQOL. Assuming that the population EVPI is £10 million, the lower 95% CI of HR would range from 1.06 (Fig. 3; panel a, solid lines) to 1.00 (Fig. 3; panel d, solid lines) in women, while from 1.07 (Fig. 3; panel j, solid lines) to 1.01 (Fig. 3; panel m, solid lines) in men. However, this scenario is associated with a large increment in HRQOL, which needed to be statistically significant in women (Fig. 3; panel a, solid lines; 95% CI: 1.24 to 1.55).

#### Confidence intervals around the HRQOL utility-ratio threshold values

The use of the exoskeleton in women would be cost-effective only if the increments in HRQOL are significant. Assuming that the population EVPI is £10 million, the 95% CI of the utility-ratio in women ranges from 1.15 to 1.43 (Fig. 3; panel a, dashed lines). However, the exoskeleton would be cost-effective if the increment in the HR of falling is no longer significant (95% CI: 0.96 to 1.89; Fig. 3 panel a, dashed lines). Conversely, the required increment in HRQOL in men did not reach the statistical significance (95% CI: 0.89 to 1.70. Figure 3; panel j, dashed lines). Similarly to women, this scenario assumed that the exoskeleton will not induce falls (HR 95% CI: 0.87 to 2.00).

Additional file 1: Appendices 11 and 12 show that the estimates for CVD patients did not drastically differ from those for dementia population. However, using the exoskeleton under a leasing agreement in patients older than 65 years will allow for larger 95% CI for HRQOL utility-ratio (Additional file 1: Appendices 11–14).

### Purchase

#### Confidence intervals around the HR threshold values

The exoskeleton could be cost-effective even if it increased the HR of falling – albeit the cost should be no more than £15,000. Although increasing the cost of the device in a single payment for the NHS and PSS will impact the 95% CI of the HR, the consequences are more evident for the HRQOL. Assuming that the population EVPI is £10 million and the cost of the exoskeleton is £15,000 (Fig. 3; panel f, solid lines), the 95% CI of the HR ranges from 1.00 to 1.94, which is paired with a utility-ratio 95% CI ranging from 1.36 to 1.68. While, if the exoskeleton priced at £17,500 (Fig. 3; panel g, solid lines), the 95% CI of the HR ranges from 0.95 to 1.85 followed by a utility-ratio 95% CI contained between 1.55 and 1.91. Given the larger 95% CIs, the trends observed in women were less evident in men (Fig. 3; panels n-p, solid lines).

#### Confidence intervals around the HRQOL utility-ratio threshold values

Smaller increments in HRQOL would be still acceptable if balanced by a decrement in the uncertainty around HR. The cost of the exoskeleton in the purchase scenarios has a prominent influence on the HR 95% CI. For example, assuming that the population EVPI is £2.5 million, the 95% CI of the HR of falling ranges from 0.68 to 0.95 and it is paired with a utility-ratio ranging from 1.76 to 1.96 (Fig. 3; panel h, dashed lines). Given the larger 95% CIs, the trends observed in women were less evident in men (Fig. 3; panels n-p, dashed lines).

Additional file 1: Appendices 15 and 17 showed no difference between the estimates for CVD and dementia patients (Additional file 1: Appendices 16 and 18). Using the exoskeleton under a purchase agreement in patients older than 65 years will allow for larger 95% CIs for HRQOL utility-ratio as well as narrower 95% CIs for the HR of falling.

## Discussion

Our study analysed the population EVPI to perform an early economic assessment of an exoskeleton preventing SHFs in high-risk populations in the UK. Based on expert opinions and assuming a maximum acceptable ICER is £20,000, further research would be justifiable in patients not older than 75 years old, especially in women. The limited number of potential male users led to smaller population EVPI, which resulted in larger acceptable uncertainty for males. This seems to suggest that the intervention may more easily satisfy cost-effectiveness requirements for male patients.

Our secondary analyses showed that the exoskeleton could still represent good value for money even if it does not meet the experts’ expectations. Given that the exoskeleton can be cost-effective even in extreme scenarios, such as those simulating an HR of falling not inferior to 1, a *placebo effect* on HRQOL may be sufficient to obtain an ICER below £20,000/QALY. Conversely, the reduction in the HR of falling should be significant if the manufacturer aspires to sell the device at more than £17,500 or seeks an indication for dementia patients older than 65 years. As noted in the base-case, sex influenced our uncertainty analyses. The confidence intervals of the parameters for male patients are typically larger than confidence intervals of the parameters for women. While, the increment of HRQOL for male should not be necessarily statistically significant, especially at larger population EVPI, women will need to have narrower confidence intervals.

The two most recent systematic reviews identified numerous techniques used during the early stages of product development [14, 22]. The use of simulation and elicitation methods are becoming the most common tools to support the investments in the early stages [22]. These reviews corroborate the importance of scenario analysis in early Health Technology Assessment (HTA) – albeit deploying this approach in early evaluations could be more computationally intensive than in standard evaluations. Given this computational burden, early HTA models usually obtain the unknown parameters, and their uncertainty, using elicitation methods. Model assumptions are then tested running a limited number of scenarios, which provide an incomplete scenario analysis. Although expert elicitation plays a prominent role in decision-making, it is not exempt from limitations, such as overconfidence and the availability of a sufficient number of experts [23]. Given the early stage of the exoskeleton development, we feared an exacerbation of these limitations. Thus, we desisted from using expert elicitation in favour of richer scenario analysis.

Although our analyses required a computationally expensive and sophisticated approach, we also believe that one of the strengths of our study is the objective results of our scenario analysis, which deliberately explored even unlikely scenarios. Running 16,848 scenarios allowed a mapping of the combinations of the exoskeleton requirements leading to an ICER below £20,000 per QALY. Two-hundred and eighty-six cost-effective scenarios were identified and, by doing so, we answered questions (i) and (ii) outlined in the background. The uncertainty around the parameters was then modelled for these 286 cost-effective scenarios, obtaining further 22,880 scenarios providing the population EVPI. Therefore, we back-calculated the maximum uncertainty allowed around the exoskeleton parameters conditional on population EVPI and, hence, answered the question (iii). The answers to questions (i-iii) are necessarily complex and should be interpreted by the decision maker alongside the future evidence on the exoskeleton performance [24]. Although answering question (iv) is beyond the objectives of this study, our results encourage the manufacturer to explore alternative business models and strategies of the production process. Reducing the cost, or the leasing fee, of the exoskeleton, will relax most of the requirements – especially on the HRQOL domain. Given that the HRQOL is likely to be a driver of the exoskeleton cost-effectiveness, engineers should take extra care of the design. For example, the exoskeleton should not interfere with patients’ day-to-day activities and should mitigate the anxiety due to a possible second fall [25]. In this respect, the decision makers will need to evaluate carefully the evidence on the impact of the exoskeleton on patients’ HRQOL. Besides the thorough scenario analysis, another strength lies in the data used for this study. In populating the model, we used administrative records from the UK population, which provided a valuable foundation for this simulation.

However, the study is not exempt from limitations. We did not account for higher costs occurring in more severe comorbid patients, such as individuals with CVD or dementia. Although the Charlson Comorbidity Index is an important cost predictor for the primary hip fracture, its impact is limited on the SHFs and, thus, it was not included in the final equations [7]. Secondly, our model did not account for spontaneous fractures and, therefore, it might underestimate the total number of SHFs. However, we anticipate this assumption is unlikely to have a material impact on our estimates since we simulated populations highly prone to fall [26]. Thirdly, we acknowledge that a meta-analysis of the HRQOL utilities, accounting for either CCI or the included comorbidities, would have been the ideal approach. Given the early stage of the exoskeleton, we deemed the disease-specific disutilities reported by Sullivan et al. to be fit for our economic evaluation [18]. Finally, we used the population EVPI, which monetised the overall uncertainty around the cost-effectiveness of the exoskeleton. Although calculating the expected value of perfect information for parameters (EVPPI) would have allowed us to estimate the impact of the uncertainty of the single parameters, EVPI was preferred because of research goals and feasibility issues. Given the early stage of the exoskeleton, we believe that the exoskeleton could be tested in a research setting aiming at goals including but not limited to the device assessment. Therefore, using EVPI provided a broader view on the monetary value of additional research compared to EVPPI. Additionally, performing an EVPPI would have been largely more computationally intensive than implementing the analysis of the population EVPI, which was already challenging because of the large number of scenarios.

## Conclusion

The current paper illustrated the use of population EVPI to identify the patients that could benefit most from the use of a new technology, the cost-effectiveness requisites of the technology and the necessary conditions for justifying future research. To inform the decision makers, we focused on these necessary conditions, while we neglected numerous scenarios where the device would still represent good value for money. Future research should fill these gaps and, thus, provide a complete picture of the EVPI function to support decisions on whether the development of technologies is worthwhile.

## Supplementary information

**Additional file 1.**

## Data Availability

The datasets generated and/or analysed during the current study are not publicly available due confidentiality arrangements but are available from the corresponding author on reasonable request.

## Abbreviations

HRQOL: Health-Related Quality of Life
SHF: Second Hip Fractures
CVD: cardiovascular disease
VOI: Value of Information
QALYs: Quality Adjusted Life Years
HES: Hospital Episode Statistics
CPRD: Clinical Practice Research Datalink
CCI: Charlson Comorbidity Index
NHS: National Health Service
HR: hazard ratio
ICER: Incremental Cost-Effectiveness Ratio
EVPI: expected value of perfect information
HTA: Health Technology Assessment
