# Correction to: Determining the cost-effectiveness requirements of an exoskeleton preventing second hip fractures using value of information

**DOI:** 10.1186/s12913-020-06007-6

**Published:** 2020-12-20

**Authors:** Stefania Manetti, Giuseppe Turchetti, Francesco Fusco

**Affiliations:** 1 Institute of Management, Scuola Superiore Sant’Anna, Pisa, Italy; 2 Department of Surgery and Cancer, St Mary’s Hospital, Imperial College London, London, UK; 3 Health Economics Research Centre, Nuffield Department of Population Health, University of Oxford, Old Road Campus, Headington, Oxford, UK; 4 Centre for Health Economics, University of York, Heslington, York, UK; 5 Department of Public Health & Primary Care, Institute of Public Health, University of Cambridge, Forvie Site, Robinson Way, Cambridge, CB2 0SR UK

**Correction to: BMC Health Serv Res 20, 955 (2020)**

**https://doi.org/10.1186/s12913-020-05768-4**

Following the publication of the original article [1], it was noted that Fig. 2 and Fig. 3 have poor resolution.

The updated figures have been included in this correction, and the original article has been corrected.

**Fig. 2:**
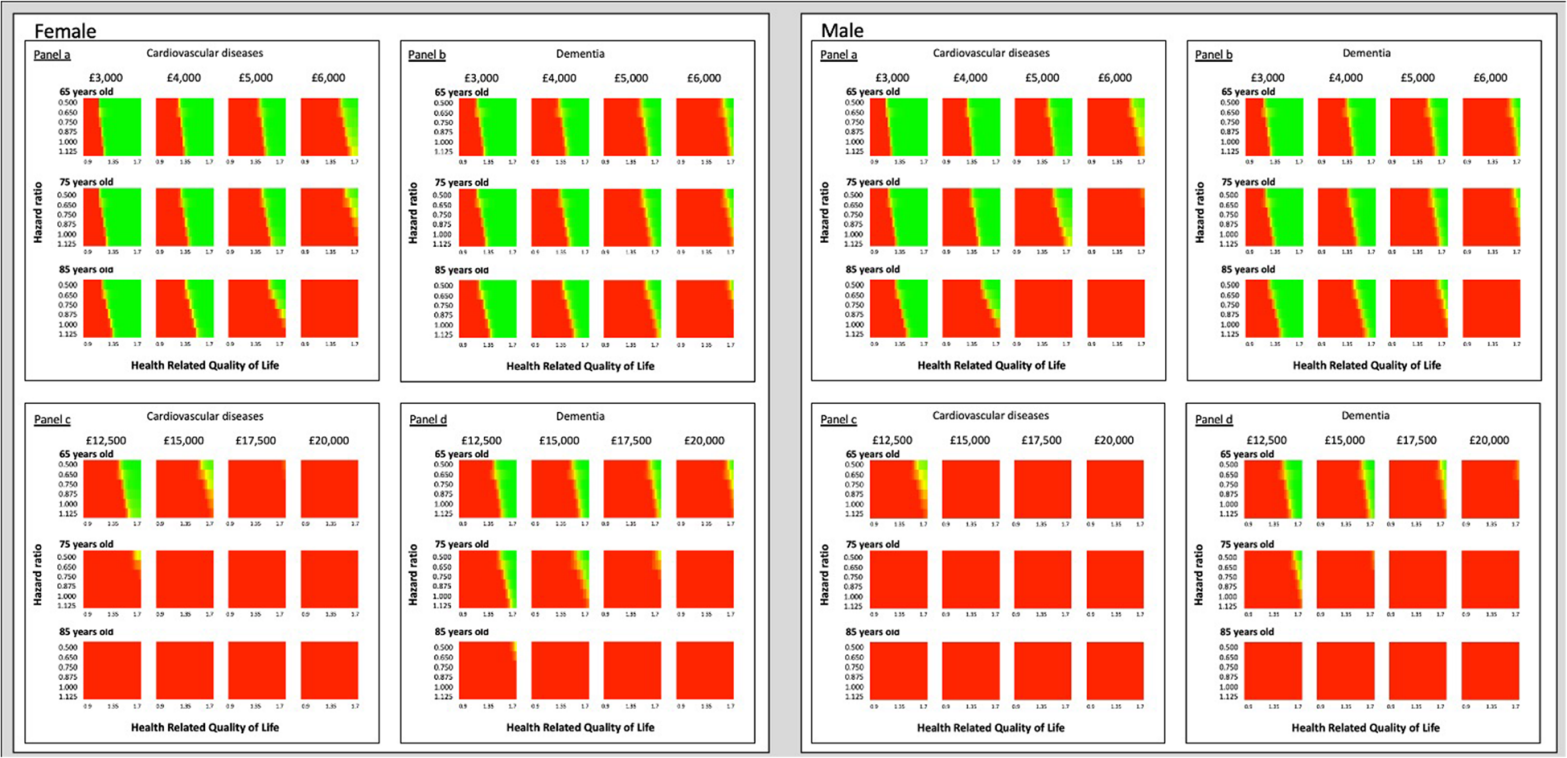
Threshold analysis: Cost-effectiveness heat map of cardiovascular and dementia hip fractured populations by sex and age. Legend: Green (cost-effectiveness probability = 1); red (cost-effectiveness probability = 0)

**Fig. 3:**
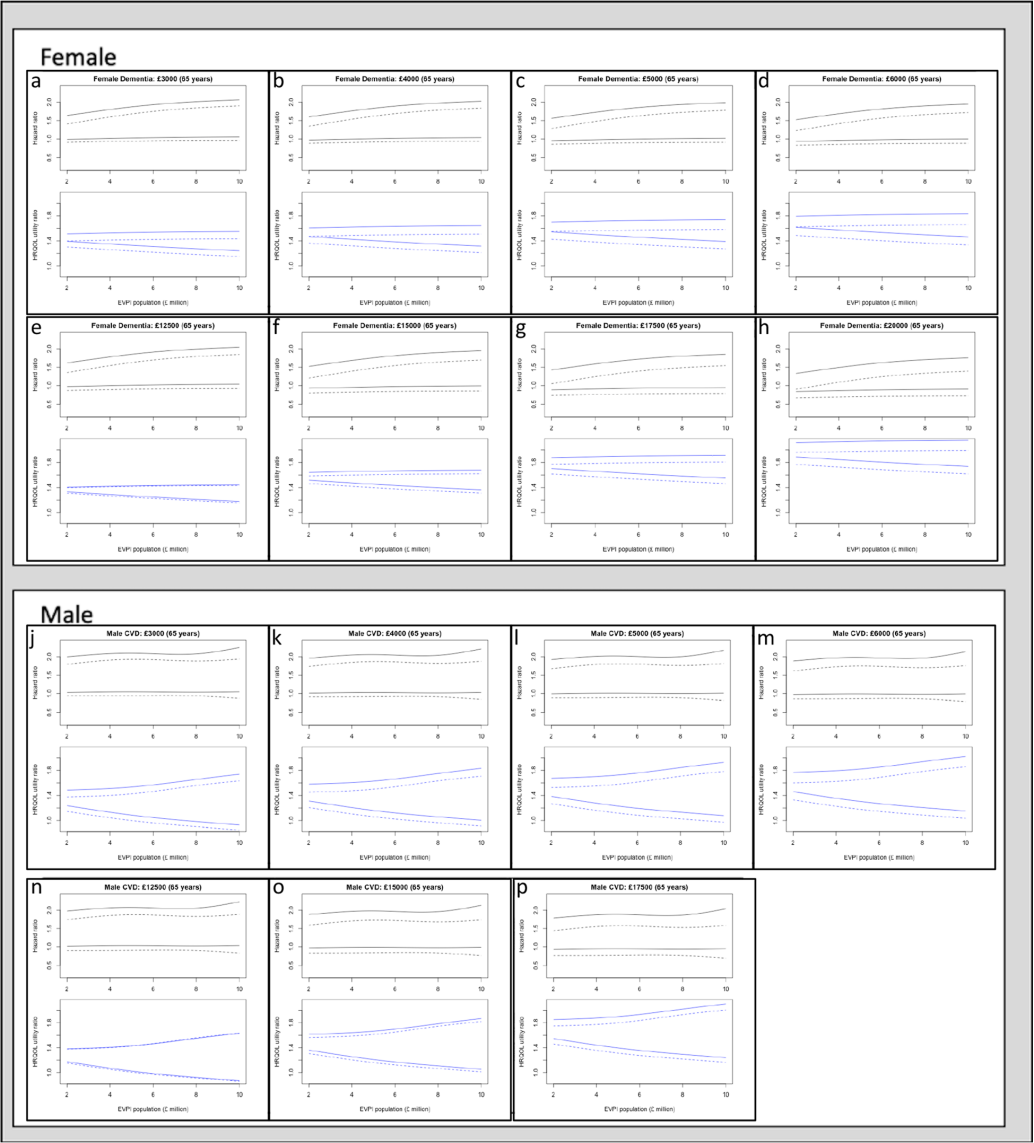
Uncertainty analysis: 95% confidence interval of HRQOL utility-ratio, 95% confidence interval of SHF hazard ratio as a function of the expected value of information at population level (£ million). Abbreviations: Health Related Quality of Life (HRQOL); Expected Value of Perfect Information (EVPI). Legend: dashed lines (HRQOL utility-ratio threshold); solid lines (hazard ratio threshold)
