# Supplementary material for: Determining the cost-effectiveness requirements of an exoskeleton preventing second hip fractures using value of information
Source: BMC Health Serv Res. 2020 Oct 15;20:955. doi: 10.1186/s12913-020-05768-4 (PMC7565816; doi:10.1186/s12913-020-05768-4)
Supplement: Supplementary file 1 — Additional file 1. [file 12913_2020_5768_MOESM1_ESM.pdf]

Appendix 1: Model schematic and allowed transitions: Panel a. discharge to care-home; Panel b. discharge to own home. (Leal et al. (1,2))

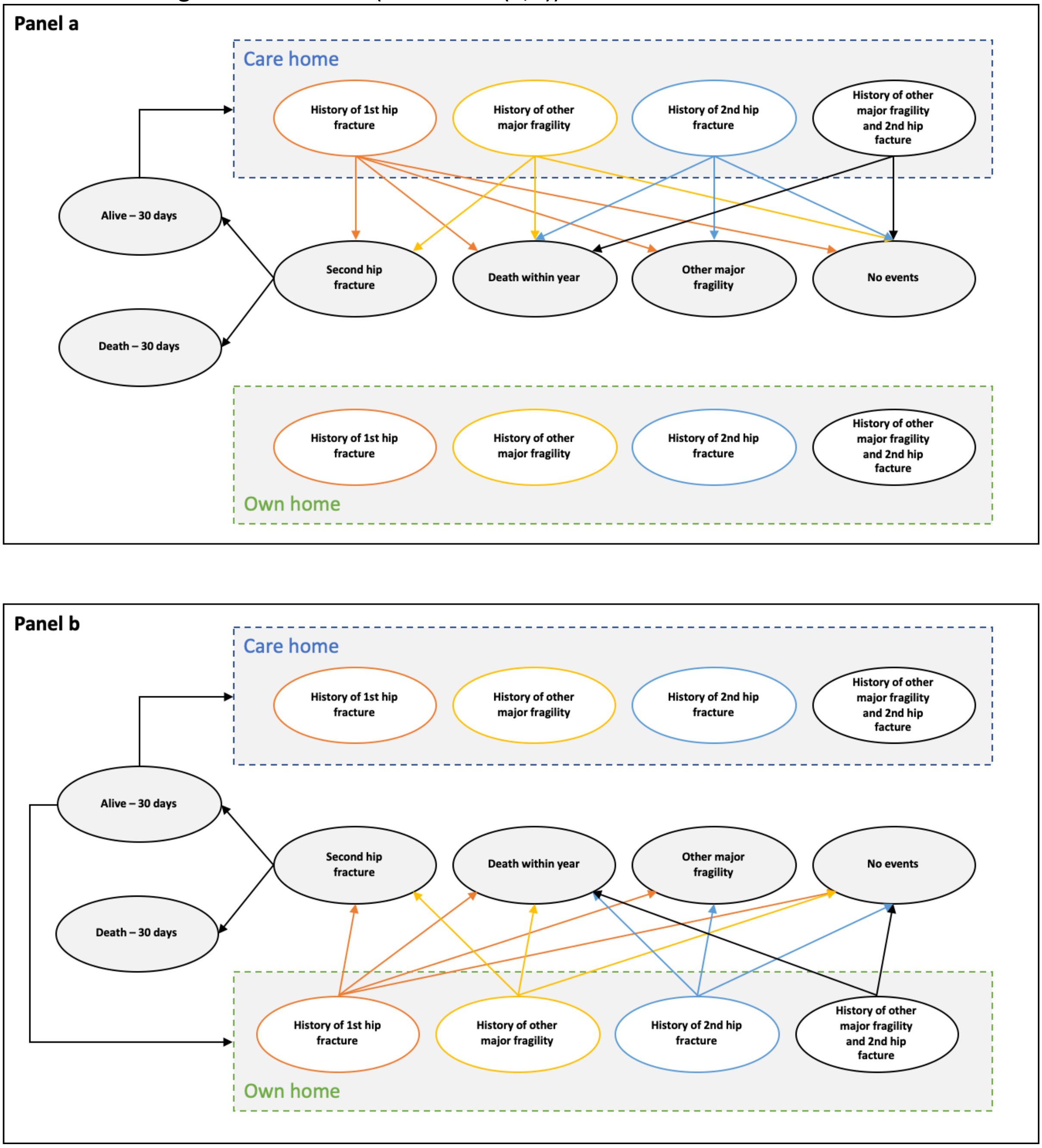

After the 1st hip fracture, patients were distributed across four health states (history of 1st hip fracture, history of other major fragilities requiring hospitalisation, history of 2nd hip fracture and history of other major fragilities and 2nd hip fracture) conditional on living in either a care-home or their own home. The model allowed patients to progress (e.g. 2nd hip fracture, other major fractures, death, no events) unless they had not reached an absorbing state, such as death. While patients living in their own home could access to a Care-home following a hospital discharge (Appendix 1: Panel b), it was assumed that patients living in a Care-home could not regress to their own home (Appendix 1: Panel a). A cycle length of one year was considered appropriate given the natural history of hip fracture patients, and half-cycle correction was performed.

Appendix 2: Risk equations estimating the admission to a care-home (Hospital Episode Statistics; Leal et al. (1,2))

**Event**	**Care-home after first hip fracture**		**Care-home after second hip fracture**	
Functional form	Logistic		Logistic	
Patients	24879		1599	
Patient-years	24879		1599	
Number of events	4869		278	
Parameters	Mean	(Standard Error)	Mean	(Standard Error)
Female	0.152	(0.040)		
Age at first hip fracture	0.173	(0.034)		
(Age at first hip fracture)^2	-0.0008	(0.0002)		
CCI score at first hip fracture	0.042	(0.011)		
Age at second hip fracture			0.030	(0.009)
Constant	-10.490	(1.398)	-4.120	(0.777)
$p>X^2$	<0.001		<0.001	

Appendix 3: Risk equations estimating all-cause mortality and events probabilities (Hospital Episode Statistics; Leal et al. (1,2))

Event	Second hip fracture		Major non-hip fracture		30-day all-cause mortality after first hip fracture		30-day all-cause mortality after second hip fracture		All-cause mortality post 30 days	
Functional form	Weibull		Weibull		Logistic		Logistic		Gompertz	
Patients	29888		29888		32989		2197		29888	
Patient-years	59740		60557		32989		2197		62907	
Number of events	2206		1464		3101		173		13008	
Parameters	Mean	(Standard error)	Mean	(Standard error)	Mean	(Standard error)	Mean	(Standard error)	Mean	(Standard error)
P	1.099	(0.018)	1.259	(0.024)						
Γ									0.012	(0.005)
Age at first hip fracture	0.042	(0.003)	0.028	(0.004)	0.075	(0.003)			0.072	(0.002)
Age at second hip fracture							0.048	(0.012)		
Female	0.117	(0.055)	0.481	(0.077)	-0.505	(0.043)	-0.623	(0.186)	-0.436	(0.020)
Care-home			0.451	(0.055)	0.236	(0.057)	0.360	(0.170)	2.092	(0.219)
CCI score at first hip fracture					0.269	(0.010)			0.655	(0.062)
Major non-hip fracture	0.371	(0.117)								
second hip fracture			0.377	(0.121)						
History of second hip fracture			0.286	(0.113)					0.246	(0.044)
History of non-hip fracture									0.152	(0.053)
Age X care-home									-0.020	(0.003)
Age X CCI score at first hip fracture									-0.006	(0.001)
Constant	-6.951	(0.244)	-6.867	(0.298)	-8.705	(0.242)	-6.264	(1.014)	-7.471	(0.143)
$p>X^2$	<0.001		<0.001		<0.001		<0.001		<0.001	

CCI score: Charlson co-morbidity index at hospital admission for 1st hip fracture (up to 3 years before); Age X care-home: interaction term for age at primary hip fracture and living in a care-home; Age X CCI: interaction term for age and CCI score at primary hip fracture.

Appendix 4: Exoskeleton and subgroup parameters used in the base-case analysis

	Value (standard error [SE] or 95% Confidence interval [95% CI])	Source
**Exoskeleton parameters**		
Hazard ratio - Second hip fracture	0.75 (SE: 0.95)	Experts' opinion: (i) Exoskeleton Manufacturers (ii) Senior consultant
Utility ratio – Exoskeleton vs usual care	1.7 (SE: NA[+])	Experts' opinion: (i) Senior consultant
Yearly cost – Exoskeleton leasing	£6,000 (SE: NA)	
**Subgroup parameters**		
Odds ratio second hip fracture - dementia	1.89 (95% CI:1.47 to 2.43)	Zhu (3)
Odds ratio second hip fracture - CVD	1.32 (95% CI: 1.02 to 1.70)	
Disutility - Cerebral Degeneration (ICD-9 331) (dementia)	0.22 (SE: 0.03)	Sullivan (4)
Disutility - Old Myocardial Infarct (ICD-9 412) (CVD)	0.04 (SE: 0.03)	Sullivan (4)
Disutility - Precerebral Occlusion (ICD-9 433) (CVD)	0.03 (SE: 0.02)	Sullivan (4)
Yearly cost – Exoskeleton leasing	£6,000 (SE: NA)	

[+]The uncertainty around the HRQOL was modelled by inflating the subgroups HRQOL uncertainty by 40%.

Appendix 5: Utility scores for hip fracture individuals (Leal et al. (1,2))

Mixed-effects model (Linear)		
No. groups	32	
No. observations	187	
Parameters	Mean	(Standard Error)
EuroQol EQ-5D	-0.181	(0.133)
Follow-up time (months)	0.017	(0.002)
Follow-up time (months^2)	0.000	(0.000)
Constant	0.622	(0.101)
Random effects	0.157	(0.037)
Prob>X^2	0.0000	

Appendix 6: Equations estimating primary care cost (Clinical Practice Research Datalink; Leal et al. (1,2))

	Year of first hip fracture		Subsequent years	
Distributional form	Gamma		Gamma	
Link function	Identity		Identity	
No. of patients	3,910		2,568	
Patient-years	3,910		7,373	
Parameters	Mean	(Standard Error)	Mean	(Standard Error)
Death - 30 days	-1197	(52)		
Death – 1 year	-689	(36)	-437	(52)
Living in care-home	126	(39)		
Major non-hip fracture			502	(264)
Constant	1251	(39)	1161	(40)

Appendix 7: Equations estimating hospital care cost (Hospital Episode Statistics; Leal et al. (1,2))

	Probability of hospitalisation in the years post 1st hip fracture		Hospitalisation costs in year of 1st hip fracture		Hospitalisation costs in subsequent years (conditional on hospitalisation)		Hospitalisation costs in year of second hip fracture (subsequent years to 1st hip fracture)	
Distributional form	Logistic		Gamma		Gamma		Gamma	
Link function			Identity		Identity		Identity	
Number of patients	18,213		30,430		8,604		1,166	
Patient-years	29,133		30,430		10,243		1,166	
Parameters	Mean	(Standard Error)	Mean	(Standard Error)	Mean	(Standard Error)	Mean	(Standard Error)
Death - 30 days of hip fracture			-5110	(129)			-3560	(589)
Death - year of hip fracture			2979	(169)			5391	(1091)
Living in care-home	0.273	(0.030)	3168	(149)	2676	(259)	1053	(520)
Age at hip fracture			24	(7)			-108	(36)
Current age					-55	(13)		
Female	-0.328	(0.032)	-1265	(129)	-1039	(268)	-1908	(762)
Major non hip fracture			5964	(707)				
Second hip fracture			10017	(635)				
History of major non hip fracture	0.364	(0.087)						
History of second hip fracture	0.383	(0.069)			993	(472)		
Constant	-0.449	(0.029)	11462	(559)	10795	(1133)	23206	(3166)

Appendix 8: Equations estimating hospital care cost (Hospital Episode Statistics; Leal et al. (1,2))

	Hospitalisation costs if major fracture occurs (subsequent years to 1st hip fracture)		Probability of hospitalisation given death		Hospitalisation costs if death occurs (conditional on hospitalisation)	
Distributional form	Gamma		Logistic		Gamma	
Link function	Identity				Identity	
Number of patients	899		9,282		5,404	
Patient-years	968		9,282		5,404	
Parameters	Mean	(Standard Error)	Mean	(Standard Error)	Mean	(Standard Error)
Living in care-home	2001	(606)	-0.209	(0.043)	896	(235)
Current age			-0.038	(0.003)	-144	(17)
Female	-2953	(1096)	-0.251	(0.053)		
Constant	11582	(1057)	3.970	(0.269)	19401	(1489)

Appendix 9. Quantile regressions – Hazard ratio (HR) threshold values. 90th quantile of the exoskeleton SHF hazard ratio and 10th quantile of the exoskeleton utility ratio.

	**HR mean (90th quantile)**	**HR lower 95% CI (90th quantile)**	**HR upper 95% CI (90th quantile)**	**Utility ratio mean (10th quantile)**	**Utility ratio lower 95% CI (10th quantile)**	**Utility ratio upper 95% CI (10th quantile)**
Intercept	2.372563(0.06409555)***	0.006614172(0.04130169)	-0.08156045(0.03905226)*	-1.641911(0.06858341)***	0.07719019(0.03196574)*	-0.001659109(0.007630735)
Dementia	0.01269568(0.00225763)***	0.000743308(0.002762654)	-0.005305762(0.002900519)	0.03430188(0.000825789)***	0.01304441(0.001332946)***	0.0445371(0.000450396)***
Age	-0.01583256(0.000918008)***	0.00000113(0.00050171)	-0.000324972(0.000431759)	0.02485985(0.000641051)***	0.001423406(0.000465321)**	-0.000530982(0.000108029)***
Leasing	-1.211417(0.06285031)***	0.01596708(0.04004696)	-0.1816009(0.03809636)***	2.326294(0.06425846)***	0.6163567(0.03355365)***	-0.08962659(0.007927308)***
Cost or annual fee exoskeleton	-0.000020129(0.000001868)***	0.000000145(0.000000793)	-0.000001832(0.000000965)	0.000089939(0.000001577)***	0.000009608(0.000000638)***	-0.000001795(0.000000239)***
+EVPI population	0.000000015(0.000000003)***	-0.000000002(0.000000004)	0.000000036(0.000000003)***	-0.000000005(0.000000001)***	-0.000000057(0.000000001)***	0.000000008(0.0000000004)***
+EVPI population'	-0.000000384(0.000000112)***	0.000000076(0.000000121)	-0.000000852(0.000000096)***	0.000000166(0.000000023)***	0.000000306(0.000000045)***	0.000000436(0.000000017)***
+EVPI population''	0.000001608(0.000000758)*	-0.000000542(0.000000734)	0.000003342(0.000000384)***	-0.000000375(0.000000118)**	-0.000000778(0.000000203)***	-0.000001211(0.000000073)***
Female	-0.06997827(0.05690123)	0.006869596(0.02094991)	-0.2924508(0.03543725)***	-0.02265739(0.004754939)***	0.09431137(0.03380005)**	-0.01278167(0.001640773)***
Hazard ratio mean		0.9016584(0.008539323)***	1.801318(0.006388525)***	0.1429219(0.01812344)***	-0.08610483(0.04787674)	0.03170197(0.01341769)*
Hazard ratio lower 95% CI				0.002480269(0.0118812)	0.02935957(0.03156402)	-0.005342361(0.008963983)
Hazard ratio upper 95% CI				-0.005844899(0.007061482)	0.06226084(0.01818662)***	-0.02230783(0.005058691)***
Utility ratio mean					0.7561814(0.008777373)***	1.111834(0.002778619)***
Utility ratio lower 95% CI						
Utility ratio upper 95% CI						
Age X Leasing	0.01593554(0.00092559)***	-0.000212933(0.000553012)	0.002402616(0.000484156)***	-0.02118178(0.000647136)***	-0.008257541(0.000467368)***	0.001072818(0.000106918)***
+EVPI population X Female	0.000000002(0.00000001)	0.000000001(0.000000004)	0.00000002(0.000000007)**	0.000000003(0.000000001)**	0.00000003(0.000000006)***	-0.000000003(0.0000000005)***
+EVPI population' X Female	0.000000282(0.000000131)*	-0.000000066(0.000000118)	0.000000504(0.000000106)***	-0.000000153(0.00000024)***	-0.000000229(0.000000059)***	-0.000000467(0.000000017)***
+EVPI population'' X Female	-0.000001465(0.000000763)	0.000000528(0.000000728)	-0.000002854(0.00000039)***	0.000000357(0.000000118)**	0.00000677(0.0000021)**	0.000001265(0.000000073)***

+ EVPI population was modelled using a restricted cubic spline * p<0.05; ** p<0.01; *** p<0.001

Appendix 10. Quantile regressions – HRQoL threshold values. 90th quantile of the exoskeleton SHF hazard ratio (HR) and 10th quantile of the exoskeleton utility ratio.

	Utility ratio mean (10th quantile)	Utility ratio lower 95% CI (10th quantile)	Utility ratio upper 95% CI (10th quantile)	HR mean (90th quantile)	HR lower 95% CI (90th quantile)	HR upper 95% CI (90th quantile)
Intercept	-0.8488005(0.1760389)***	0.1288419(0.02204823)***	-0.000611688(0.0071346)	3.021679(0.05013094)***	0.1341481(0.02611658)***	-0.08244105(0.04589034)
Dementia	0.03296519(0.001348869)***	0.01430038(0.001442205)***	0.04324907(0.000437346)***	-0.008660008(0.00445755)	-0.0170914(0.003956075)***	0.01528393(0.003997553)***
Age	0.01959684(0.001786586)***	0.000623664(0.000309559)*	-0.000461134(0.000100972)***	-0.03195063(0.000740772)***	-0.000913539(0.000317579)**	-0.000494706(0.000630476)
Leasing	1.647896(0.1653726)***	0.5511969(0.02387922)***	-0.07493805(0.007468176)***	-2.725846(0.05879485)***	0.14974(0.0304572)***	-0.3354519(0.05142692)***
Cost or annual fee exoskeleton	0.000068602(0.000003165)***	0.000008006(0.000000554)***	-0.000001307(0.000000227)***	-0.000092546(0.000002319)***	0.000000038(0.0000011)	-0.000001609(0.000001625)
⁺EVPI population	-0.000000002(0.000000001)	-0.000000054(0.000000001)***	0.000000006(0.0000000003)***	0.000000035(0.000000004)***	-0.000000032(0.000000004)***	0.000000054(0.000000003)***
⁺EVPI population'	0.000000156(0.000000057)**	0.000000222(0.000000042)***	0.000000485(0.000000016)***	-0.000000545(0.000000127)***	0.000000433(0.0000001)***	-0.000000784(0.0000001)***
⁺EVPI population''	-0.000000392(0.000000266)	-0.000000413(0.000000174)*	-0.000001362(0.000000077)***	0.000001216(0.000000996)	-0.000001773(0.000000716)*	0.000003131(0.000000424)***
Female	-0.05578767(0.01705624)**	0.09428919(0.0350822)**	-0.006787809(0.001976852)***	-0.0741329(0.03078492)*	0.04621409(0.01158984)***	-0.3195769(0.02158617)***
Hazard ratio mean					0.8741843(0.008400658)***	1.791385(0.006090047)***
Hazard ratio lower 95% CI						
Hazard ratio upper 95% CI						
Utility ratio mean		0.7754441(0.008192446)***	1.10603(0.002490434)***	0.8252195(0.09200904)***	-0.08439634(0.06360885)	0.09042186(0.07768166)
Utility ratio lower 95% CI				0.2188021(0.03113367)***	-0.2093453(0.03212379)***	0.2659725(0.0278694)***
Utility ratio upper 95% CI				-0.1242868(0.05698858)*	0.2659139(0.05091918)***	-0.3229142(0.05385991)***
Age X Leasing	-0.015284(0.001762406)***	-0.007455608(0.000315974)***	0.000905967(0.000099134)***	0.0290206(0.000786563)***	-0.002207222(0.000351823)***	0.004647072(0.00065901)***
⁺EVPI population X Female	0.000000006(0.000000003)	0.000000028(0.000000006)***	-0.000000003(0.0000000005)***	-0.000000006(0.000000006)	0.000000017(0.000000003)***	0.00000001(0.000000005)*
⁺EVPI population' X Female	-0.00000018(0.000000063)**	-0.000000151(0.000000059)**	-0.000000508(0.000000016)***	0.00000038(0.000000134)**	-0.000000366(0.000000099)***	0.000000419(0.000000105)***
vEVPI population'' X Female	0.000000424(0.000000268)	0.00000032(0.000000183)	0.000001405(0.000000078)***	-0.000000979(0.000000997)	0.000001681(0.000000714)*	-0.000002619(0.000000426)***

⁺ EVPI population was modelled using a restricted cubic spline * p<0.05; ** p<0.01; *** p<0.001

Appendix 11. Leasing – Female (CVD). Expected value of information at population level,95% CI of HRQoL utility ratio and 95% CI of SHF hazard ratio.

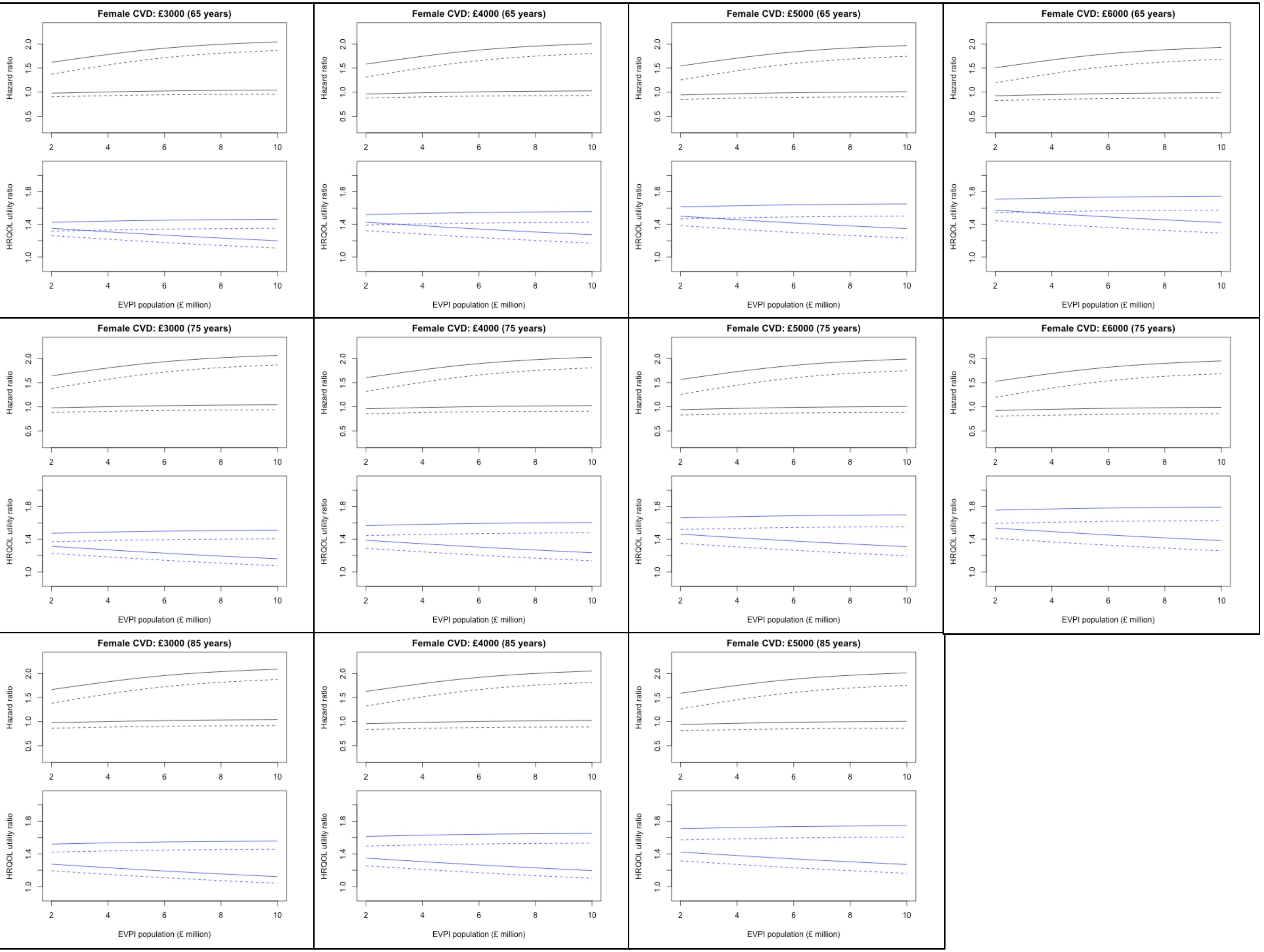

Legend: dashed lines (HRQoL utility ratio threshold); solid lines (Hazard ratio threshold)

Appendix 12. Leasing – Male (CVD). Expected value of information at population level,95% CI of HRQoL utility ratio and 95% CI of SHF hazard ratio.

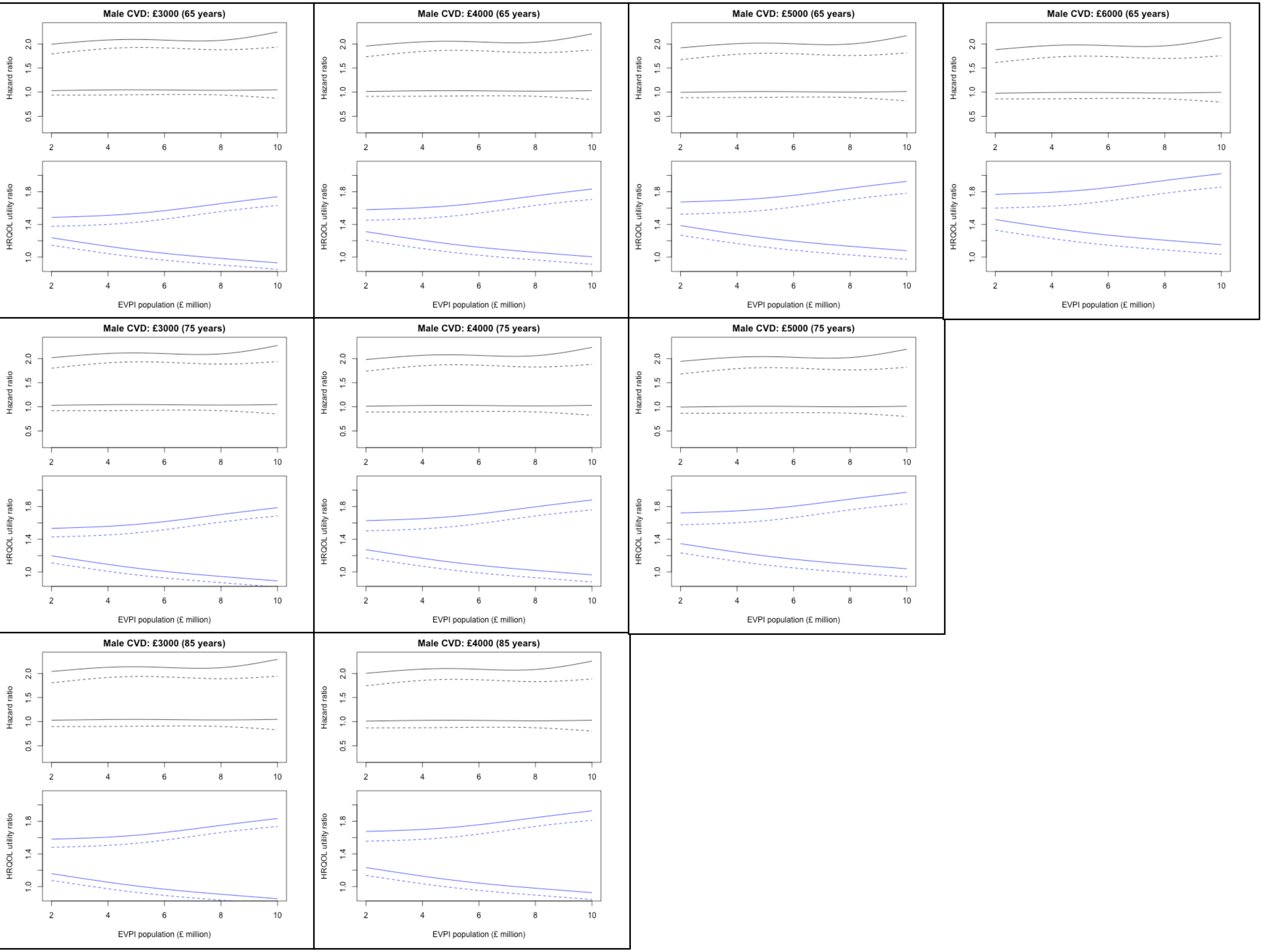

Legend: dashed lines (HRQoL utility ratio threshold); solid lines (Hazard ratio threshold)

Appendix 13. Leasing – Female (Dementia). Expected value of information at population level,95% CI of HRQoL utility ratio and 95% CI of SHF hazard ratio.

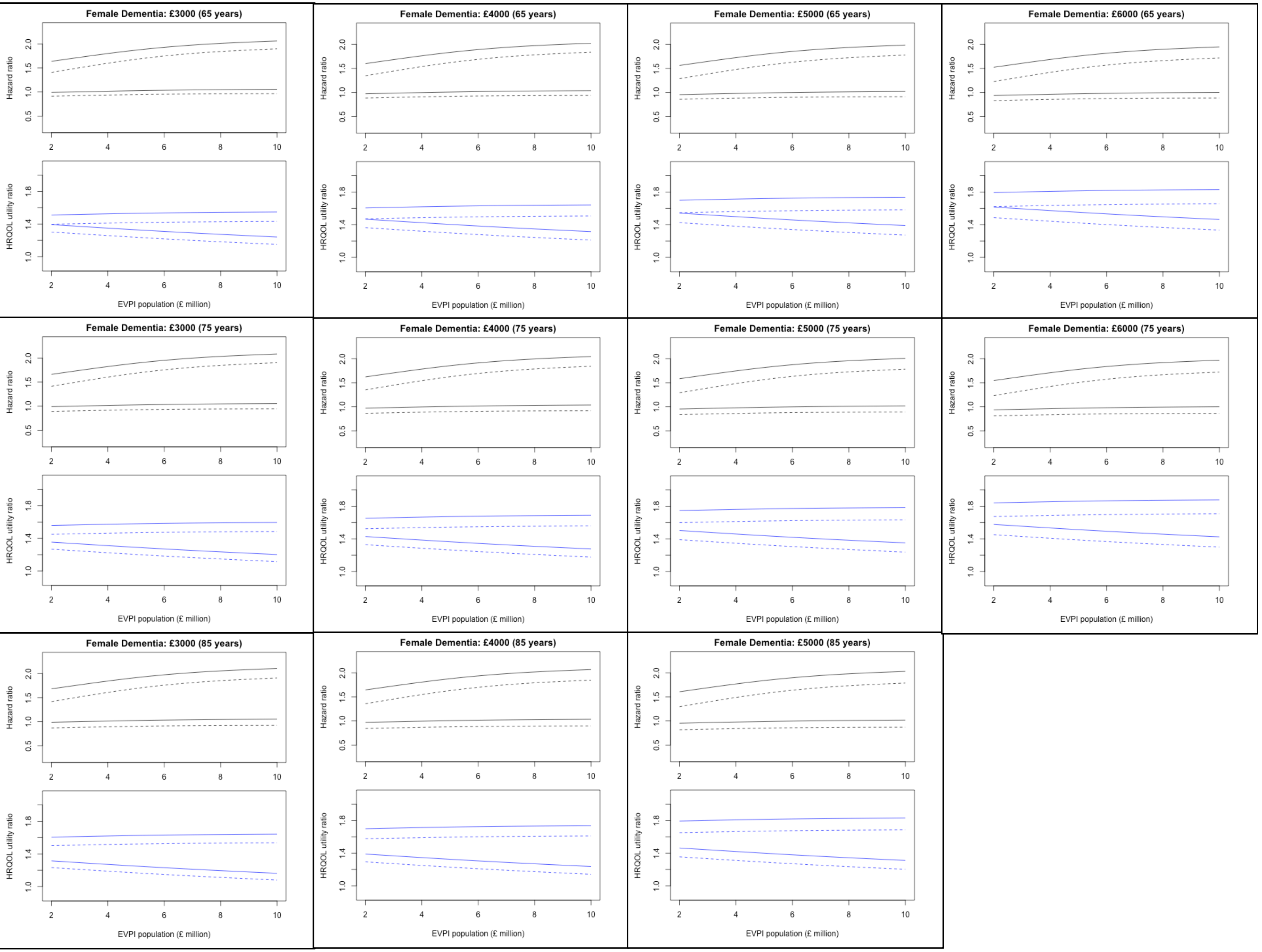

Legend: dashed lines (HRQoL utility ratio threshold); solid lines (Hazard ratio threshold)

Appendix 14. Leasing – Male (Dementia). Expected value of information at population level,95% CI of HRQoL utility ratio and 95% CI of SHF hazard ratio.

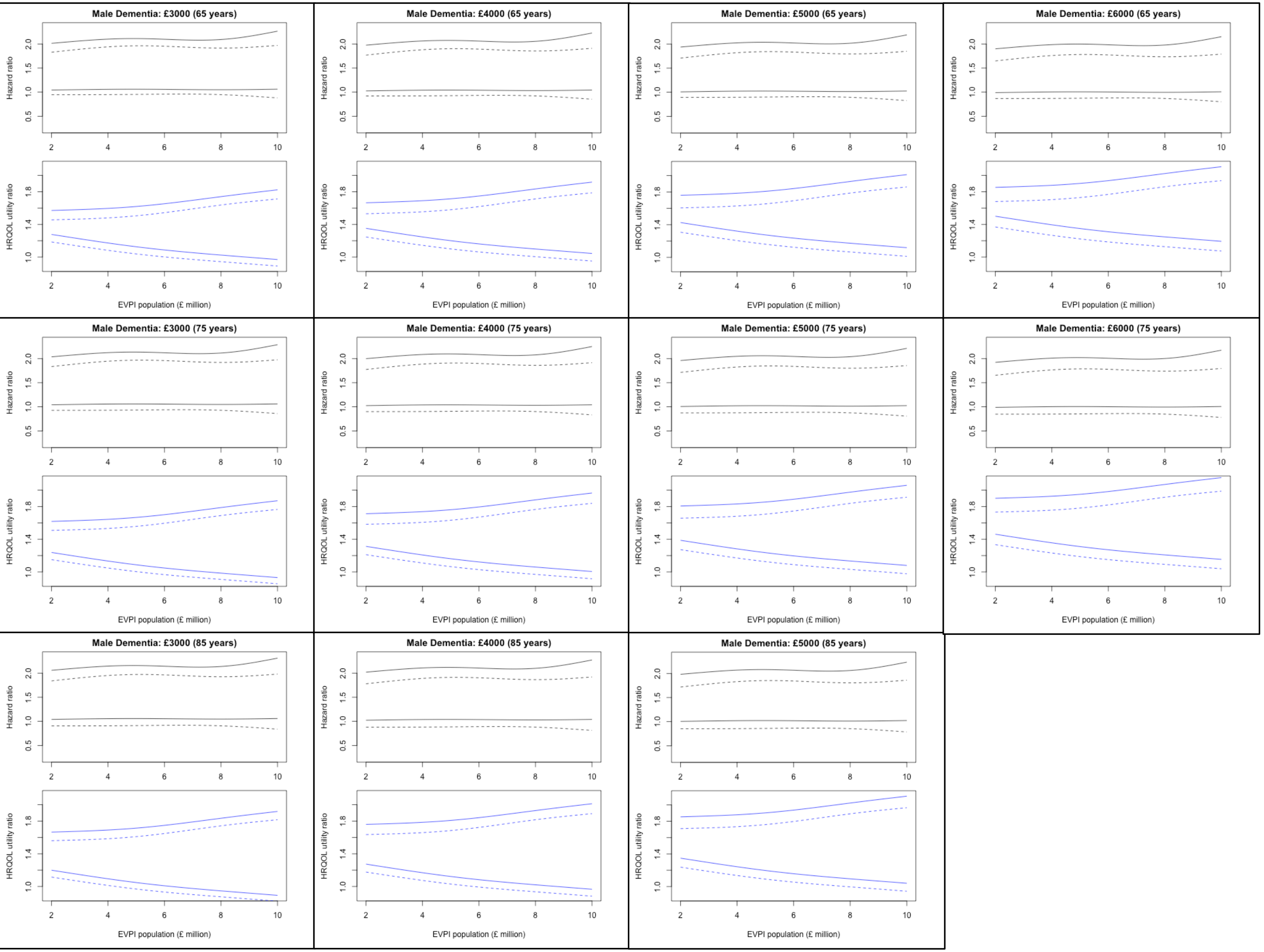

Legend: dashed lines (HRQoL utility ratio threshold); solid lines (Hazard ratio threshold)

Appendix 15. Purchase – Female (CVD). Expected value of information at population level,95% CI of HRQoL utility ratio and 95% CI of SHF hazard ratio.

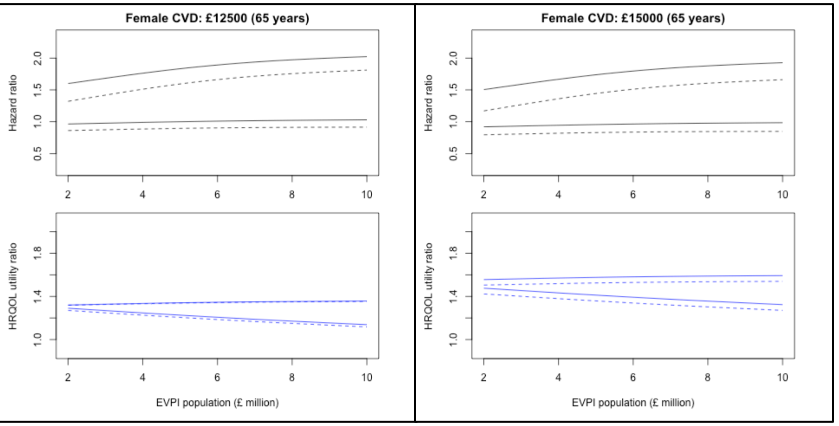

Legend: dashed lines (HRQoL utility ratio threshold); solid lines (Hazard ratio threshold)

Appendix 16. Purchase – Female (Dementia). Expected value of information at population level,95% CI of HRQoL utility ratio and 95% CI of SHF hazard ratio.

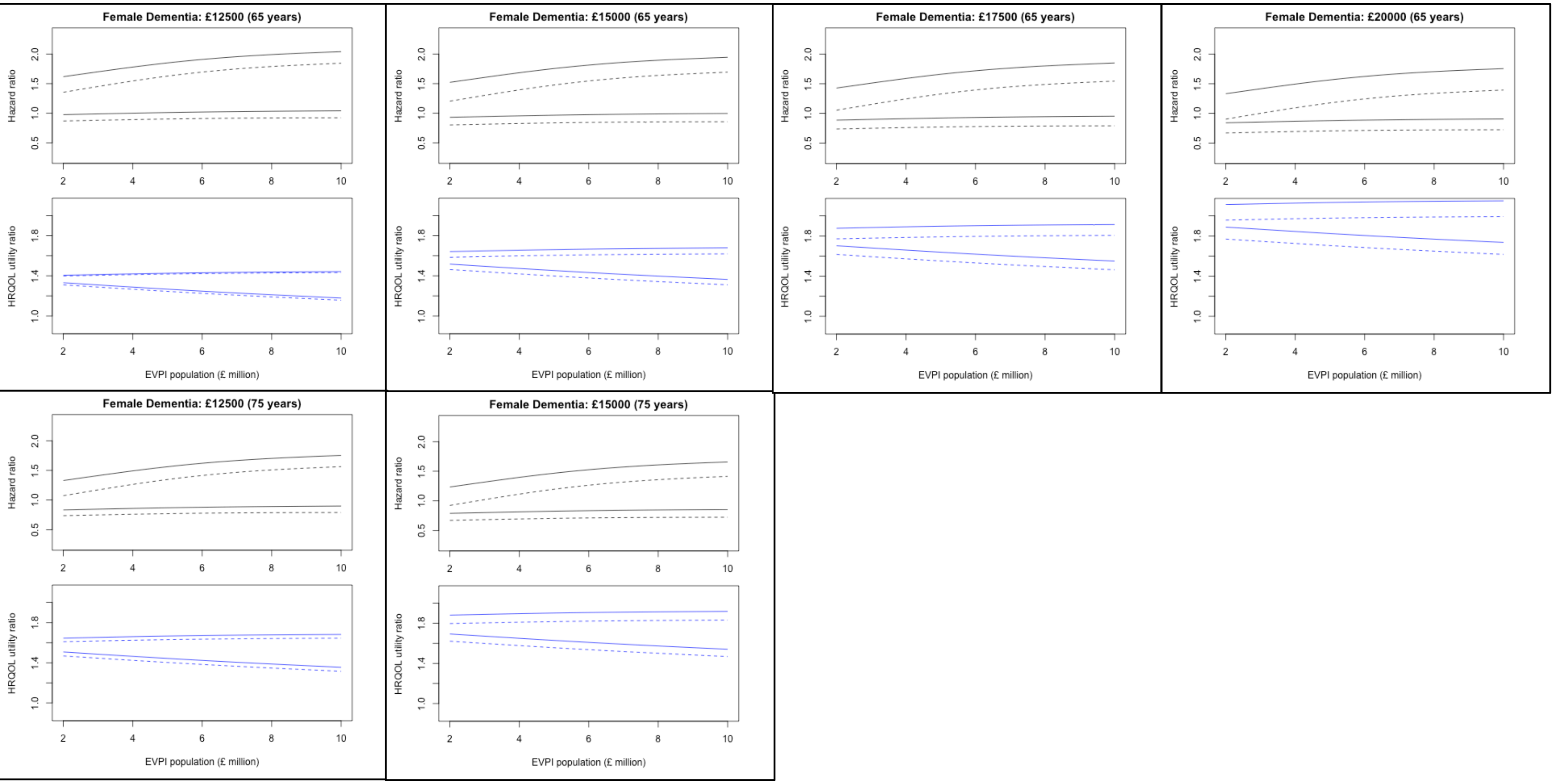

Legend: dashed lines (HRQoL utility ratio threshold); solid lines (Hazard ratio threshold)

Appendix 17. Purchase – Male (CVD). Expected value of information at population level,95% CI of HRQoL utility ratio and 95% CI of SHF hazard ratio.

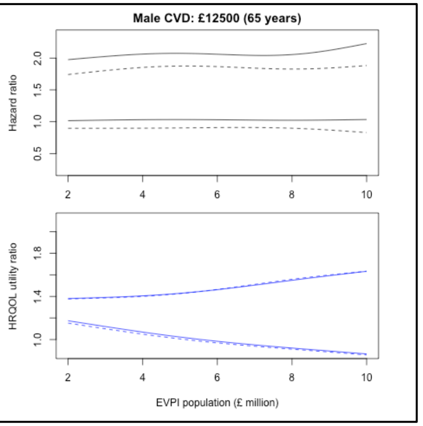

Legend: dashed lines (HRQoL utility ratio threshold); solid lines (Hazard ratio threshold)

Appendix 18. Purchase – Male (Dementia). Expected value of information at population level,95% CI of HRQoL utility ratio and 95% CI of SHF hazard ratio.

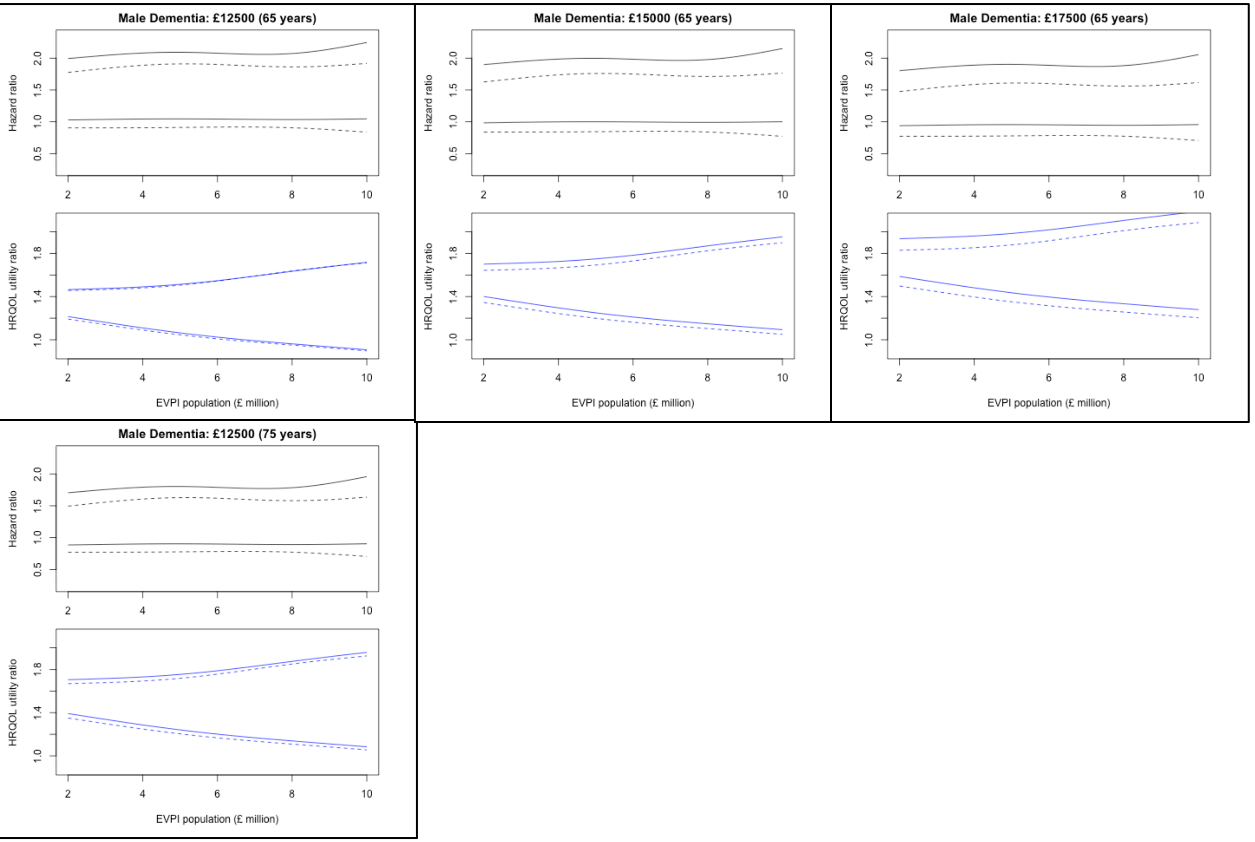

Legend: dashed lines (HRQoL utility ratio threshold); solid lines (Hazard ratio threshold)

References

1. Leal J, Gray AM, Prieto-Alhambra D, Arden NK, Cooper C, Javaid MK, et al. Impact of hip fracture on hospital care costs: a population-based study. Osteoporosis international : a journal established as result of cooperation between the European Foundation for Osteoporosis and the National Osteoporosis Foundation of the USA. 2016;27(2):549-58.
2. Leal J, Gray AM, Hawley S, Prieto-Alhambra D, Delmestri A, Arden NK, et al. Cost-Effectiveness of Orthogeriatric and Fracture Liaison Service Models of Care for Hip Fracture Patients: A Population-Based Study. Journal of bone and mineral research : the official journal of the American Society for Bone and Mineral Research. 2017;32(2):203-11.
3. Zhu Y, Chen W, Sun T, Zhang Q, Cheng J, Zhang Y. Meta-analysis of risk factors for the second hip fracture (SHF) in elderly patients. Archives of gerontology and geriatrics. 2014;59(1):1-6.
4. Sullivan PW, Slejko JF, Sculpher MJ, Ghushchyan V. Catalogue of EQ-5D scores for the United Kingdom. Medical decision making : an international journal of the Society for Medical Decision Making. 2011;31(6):800-4.
